# The Effect of a Patient Portal With Electronic Messaging on Patient Activation Among Chronically Ill Patients: Controlled Before-and-After Study

**DOI:** 10.2196/jmir.3462

**Published:** 2014-11-19

**Authors:** Iiris Riippa, Miika Linna, Ilona Rönkkö

**Affiliations:** ^1^Aalto UniversityEspooFinland; ^2^National Institute for Health and WelfareHelsinkiFinland; ^3^Hämeenlinnan Terveyspalvelut Public UtilityHämeenlinnaFinland

**Keywords:** chronic illness, patient activation, self-management, diagnosis

## Abstract

**Background:**

It has been suggested that providing patients with access to their medical records and secure messaging with health care professionals improves health outcomes in chronic care by encouraging and activating patients to manage their own condition.

**Objectives:**

The aim was to evaluate the effect of access to a patient portal on patient activation among chronically ill patients. Further, the relationship between temporal proximity of a severe diagnosis and patient activation were assessed.

**Methods:**

A total of 876 chronically ill patients from public primary care were allocated to either an intervention group receiving immediate access to a patient portal that included their medical records, care plan, and secure messaging with a care team, or to a control group receiving usual care. Patient Activation Measure (PAM) at baseline and at 6-month follow-up was obtained from 80 patients in the intervention group and 57 patients in the control group; thus, a total of 137 patients were included in the final analysis.

**Results:**

No significant effect of access to patient portal on patient activation was detected in this study (*F*_1,133_=1.87, *P*=.17, η^2^=0.01). Patients starting at a lower level of activation demonstrated greater positive change in activation compared to patients starting at higher levels of activation in both the intervention and control groups. Further, patients diagnosed with a severe diagnosis during the intervention showed greater positive change in patient activation compared to patients whose last severe diagnosis was made more than 2 years ago. The results also suggest that the intervention had greatest effect on patients starting at the highest level of patient activation (difference in change of patient activation=4.82, *P*=.13), and among patients diagnosed within a year of the intervention (difference in change of patient activation=7.65, *P*=.12).

**Conclusions:**

Time since last severe diagnosis and patient activation at baseline may affect changes in patient activation, suggesting that these should be considered in evaluation of activating chronic care interventions and in the specification of possible target groups for these interventions. This may be relevant in designing services for a heterogeneous group of patients with a distinct medical history and level of activation.

## Introduction

Approximately 40% of the population in Europe and the United States suffer from at least 1 chronic disease, and this number is expected to grow [1]. Such conditions currently account for between 70% and 80% of health care costs in these regions. To ease the burden of growing demand and restricted resources, health care providers have begun developing and implementing practices to engage the most underutilized asset of health care—the patient—in the care process. By engaging chronically ill patients in monitoring and managing their health, care providers can shift to the patients some aspects of the work previously performed by professionals. However, diminishing resource use is not the only expected benefit of activating patients. Activated patients who are knowledgeable, skilled, and confident in the self-management of their condition are shown to engage in preventive behavior by following care recommendations and a healthy lifestyle [2-4]. Engaging patients in self-management of their conditions may, therefore, also improve health outcomes and quality of care.

A growing body of research shows that sharing information regarding the state and goals of care and improving access to communication with a health care professional can strengthen a patient’s active role in the management of their own condition [5-7]. Along with the recent progress in information technology, new channels of communication between the patient and the professional, and opportunities for the care providers to effectively share information on the care process with the patient have emerged. One of the outputs of this progress is the electronic patient portal which offers the patient access to the health information that is documented and managed by a health care institution [7,8].Typically, the available information consists of the medical records of the patient, but other services and applications, such as electronic messaging with a health care professional, medication refills, and access to medical information, may also be offered [8]. Although previous studies [9,10] have shown positive effects of access to electronic patient portals on patient activation (knowledge, skills, and confidence in managing one’s condition), further empirical evidence is still required [2,8]. Moreover, little is known of the contextual factors that may promote or diminish the effect of patient portals and other self-management interventions.

We address 2 essential factors that may promote or dilute the effect of self-management interventions; namely, the level of a patient’s activation when entering an intervention and the temporal proximity of a diagnosis. Patient activation may have an impact on self-management intervention outcomes, especially when the intervention requires some level of patient participation. Temporal proximity of a diagnosis is related to a patient’s perception of their health and the consequential interest in managing their health. The health belief model by Rosenstock and colleagues [11] hypothesizes that a threat perceived by the patient of falling ill motivates health-related action if the patient believes that they may reduce the perceived threat. A strong indication of falling ill, even when the symptoms are mild, is the diagnosis made by a health care professional: “...from a patient’s perspective, [a diagnosis] is the starting point for an altered life situation” [12]. In this study, we analyzed the independent effect of time since a patient’s new diagnosis on patient activation and the moderating effect that the temporal proximity of a diagnosis may have on the activating effect of a patient portal. The more severe the disease is, the more its onset will affect patients’ attitudes toward managing their health [13]. In this study, we limited the analysis of time since diagnosis to diagnoses considered severe (eg, cancer) in contrast to diagnoses considered less severe (eg, hypertension).

This paper describes the results of a controlled before-and-after study in which the effect on patient activation of a simple patient portal with access to personal clinical information and electronic messaging with clinicians was examined. In addition, we assessed the effects of patient activation at baseline and time since severe diagnosis on change in patient activation. The study was conducted among the chronically ill patients in public primary care in a medium-sized town in Finland (approximately 68,000 citizens). Because it has been suggested that the benefits of a patient portal apply to all regular primary care customers, we did not restrict study participation on the basis of specific diagnoses, but instead based it on a professional’s perception of the chronic, but treatable, nature of a patient’s condition.

## Methods

### Study Setting, Participants, and the Intervention

This was a controlled before-and-after study conducted in Finnish public primary care. Patients visiting 1 of the 10 health centers in the town of Hämeenlinna during the recruitment phase from October 2011 to March 2012 were considered potential study participants. To study the impact of a patient portal among those most likely to become users of such a service in the future, the following eligibility criteria were applied: (1) age at least 18 years, (2) at least 2 treatable health conditions assessed by a health professional, (3) bank identifiers (electronic credentials for online authentication provided by their bank) and access to the Internet, (4) willing and able, both according to themselves and to a health care professional, to engage in using the portal.

**Figure 1:**
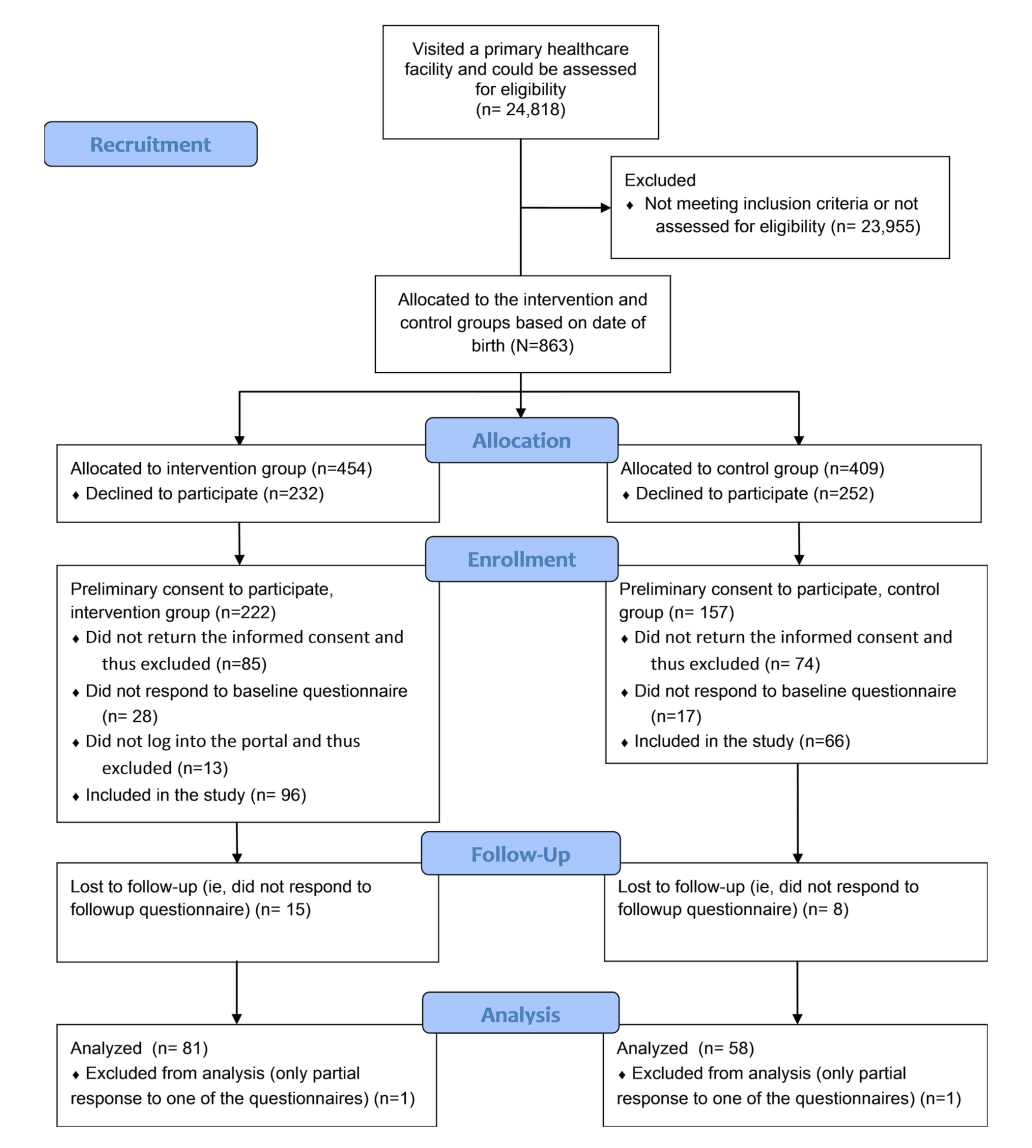
Patient flow.

The eligible patients were approached during their visits to primary health care facilities. The nurses and doctors were advised to consider each patient as a potential participant. Once a patient was found eligible, invited to participate, and showed interest in taking part in the study, they were allocated either to the intervention group or the control group on the basis of their date of birth. Patients born on odd dates were assigned to the intervention group and patients born on even dates were assigned to the control group. The intervention group received immediate access to the patient portal and participants in the control group were to receive delayed portal access after 6 months. Ethical approval was granted by the ethical board of the local authority (Pirkanmaa Hospital District). Patients who returned the informed consent to participate were included in the study, whereas patients who did not return the informed consent were considered to have declined to participate (Figure 1).

**Figure 2:**
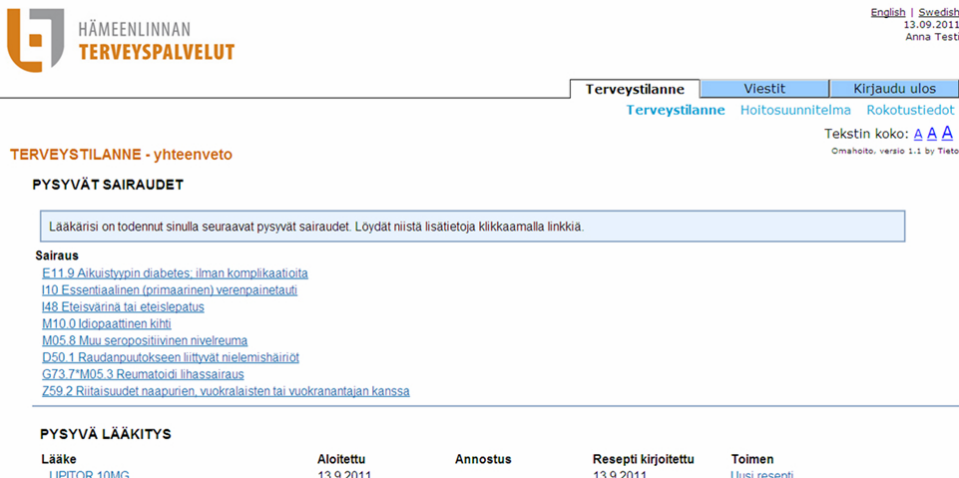
Screenshot of the patient portal.

Once a patient enrolled in the study, they formed a care plan together with a health care professional. The plan was personally tailored for each patient to holistically care for their health and to involve them in the planning of their own health care. Although a care plan was created for all study participants, only the patients in the intervention group were given online access to their care plan through the portal. Patients in the control group received a printed copy of their plan. Other features of the patient portal were access to (1) customer’s own patient records provided and maintained by the health care provider with diagnoses of chronic illnesses and permanent medication prescriptions (Figure 2), (2) laboratory results with statements from a health care professional, (3) vaccination history, and (4) electronic messaging with a health care professional. The names of diagnoses, medicines, and laboratory results were linked to relevant additional information in the online medical information service, Health Library [14], administered by The Finnish Medical Society, Duodecim. The users could visit the portal through the care provider’s webpages. For secure identification, the patient used their bank identifiers to sign in. Whenever the customer received a message or a laboratory result through the portal, a text message reminder was sent to their mobile phone. A reminder was also sent if changes to their next follow-up appointment were made.

### Materials

Patient activation was studied through the short form of Patient Activation Measure (PAM13) created by Hibbard and colleagues [15]. PAM13 assesses a patient’s knowledge of their diseases, skills to self-manage their disease, and self-confidence in their abilities to manage their disease [15]. The concept of patient activation draws on psychological theories of health locus of control [16], self-efficacy in self-managing behaviors [17], and readiness to change health-related behaviors [18], but it also incorporates competency elements specifically related to the self-management of a chronic illness [19]. The measure was developed using Rasch analysis and has been validated through several studies [3,4,20]. Increases in patient activation score have been shown to be followed by improved health behaviors [3]; thus, the measure can be used as an intermediate outcome measure for self-management interventions [2].

The PAM13 instrument consists of 13 statements, such as “When all is said and done, I am the person who is responsible for taking care of my health” (see Multimedia Appendix 1). Respondents answer the items with degrees of agreement or disagreement. The raw PAM scores (range 13-52) were linearly converted to activation scores ranging from zero (lowest activation) to 100 (highest activation) following established PAM methodology [19]. The converted PAM score was further categorized to 4 developmental levels of activation described earlier [21]. In previous studies, high PAM scores have been associated with high quality, cost-effective care [4], and an increase in activation score has also been shown to result in improved self-management behavior [3], better health outcomes [2], and a decrease in use of hospital services [2]. To collect the responses to the PAM13 questionnaire, an email with a link to an online questionnaire was sent to the participants at baseline and at 6-month follow-up.

Because a Finnish translation of PAM13 has not been used in previous studies, the translation was conducted in collaboration with an expert panel of 3 researchers with expertise in health service research. An independent Finnish translator first translated the questionnaire to Finnish, after which each member of the expert panel made their translations of the instrument. Discrepancies were discussed and a single translation of the PAM13 was agreed upon.

Diagnoses of the participants from 5 years before the intervention were gathered from the electronic patient records to examine the temporal proximity of a diagnosis. Because the effect of diagnosis on patient activation is assumed to depend on diagnosis severity [13], we defined a list of severe chronic diagnoses using the Charlson Comorbidity Index (CCI). The CCI is a widely used system for characterizing patient comorbidities drawing on information regarding 17 chronic medical conditions [22] (Multimedia Appendix 2).

### Statistical Analysis

Independent sample *t* tests for continuous variables and chi-square tests for categorical variables were used to examine the differences between the intervention and control groups at baseline. An analysis of covariance (ANCOVA) with patient activation score at baseline as a covariate was used to assess the effect of patient portal access on patient activation score at 6-month follow-up.

To examine the main effect of (1) patient activation level at baseline and (2) severe diagnosis proximity on the change in activation score, we used post hoc tests for group comparisons. In the post hoc tests, we employed the Tukey honestly significant difference (HSD) method to compare the change in patient activation between groups with different times since severe diagnosis (0-1 year, 1-2 years, over 2 years, severe diagnosis during the intervention, and no severe diagnoses), and between groups with different levels of patient activation at baseline (1-2, 3, and 4).

To test the moderating effect of (1) patient activation level at baseline and (2) severe diagnosis proximity on intervention outcome, we used linear regression modeling. Estimates (linear predictions) for changes in patient activation are presented for each category of the moderating variables.

To verify the reliability of the translated Finnish PAM13 instrument, we analyzed item response rate, internal consistency (Cronbach alpha), and item-rest correlations at both baseline and follow-up. All statistical analyses were performed using Stata version 13 (StataCorp LP, College Station, TX, USA). We used a CHARLSON Stata module by Stagg [23] to identify the CCI conditions from patient records.

## Results

### Descriptive Characteristics

A total of 24,818 unique patients visited the health care facilities during the recruitment phase and could be assessed for eligibility. Of the assessed patients, 863 met the inclusion criteria and were allocated to intervention and control groups. In the end, informed consent and responses to baseline and follow-up questionnaires were obtained from 80 patients in the intervention group and 57 patients in the control group; thus, a total of 137 patients were included in the final analysis (Table 1).

None of the differences in patients’ baseline characteristics were statistically significant. There were slightly fewer women in the control group (45.6%, 26/57) than in the intervention group (56.3%, 45/80). More patients in the intervention group had a CCI of zero (52.5%, 42/80) than in the control group (47.4%, 27/57); accordingly, a greater number of patients in the control group (21.1%, 12/57) had a CCI of 2 than patients in the intervention group (15.0%, 12/80). In addition, more patients in the control group had diagnosed hypertension (36.8%, 21/57) than patients in the intervention group (27.5%, 22/80). The mean age and the baseline score for mental health were similar in both groups as were the proportions of patients with diabetes and hypercholesterolemia.

**Table 1:** Baseline characteristics of study participants (N=137).

Characteristic		Portal access (n=80)	Control (n=57)	*t*_135_	χ^2^(*df*)	*P* value
Age (years), mean (SD)		61 (9)	63 (10)	–0.8		.40
Female, n (%)		45 (56.2)	26 (45.6)		1.5 (1)	.22
**Diagnosis, n (%)**						
	Type 1 or 2 diabetes^a,b^	32 (40.0)	22 (38.6)		0.0 (1)	.87
	Hypertension^a,c^	22 (27.5)	21 (36.8)		1.3 (1)	.25
	Hypercholesterolemia^a,d^	37 (46.3)	24 (42.1)		0.2 (1)	.63
**Charlson comorbidity index, n (%)**					0.9 (2)	.64
	0	42 (52.5)	27 (47.4)			
	1	26 (32.5)	18 (31.6)			
	2	12 (15.0)	12 (21.1)			

^a^From before the beginning of the intervention.

^b^ ICD10 codes E10-E14 or ICPC codes T89-T90.

^c^ ICD10 codes I10-I15 or ICPC codes K85-K87.

^d^ ICD10 codes E78 or ICPC T93.

### Validation of PAM

To verify the psychometric properties of the translated instrument, internal consistency and item-rest correlations were examined at both baseline and follow-up (Multimedia Appendix 3).

The item response was high, with at most 0.7% (1/137) missing values at baseline and 1.5% (2/137) at follow-up. Question 12 was scored as “not applicable” by 9.5% (13/137) of the participants at baseline and by 12.4% (17/137) at follow-up. The overall mean PAM score in this Finnish sample was 63.59 (SD 15.00) at baseline and 63.55 (SD 14.80) at follow-up, and these are similar to the Danish (64.2) [24] and Dutch (61.3) [25] mean scores.

Internal consistency was assessed as the Cronbach alpha for the sum scale, which was .87 at baseline and .86 for the follow-up sample. These are similar to the Danish (.89) [24] and Dutch (.88) [25] versions and considered to be good levels of internal consistency.

Item-rest correlation per item to the sum scale was .32 to .73 at baseline and .33 to .70 at follow-up. For several items, these values were only moderate (≤.50), which indicates that they may not be absolutely true to 1 dimension.

### Use of the Patient Portal Functionalities

The view to patient’s own health information containing diagnoses, medication prescriptions, and laboratory results was the starting page encountered by the patient once they logged in to the portal. On average, this information was viewed 10.8 times per patient during the 6-month study period. The second most popular feature of the portal, used 3.2 times on average, was viewing one’s personal care plan. Patients sent 1.5 messages to their care team and viewed their vaccination record 1.3 times on average. Only 0.3 prescription renewals, on average, were made through the portal during first year after access (Table 2).

**Table 2:** Mean use of patient portal functionalities per patient in the intervention group (n=80) during the 6-month study period.

Functionality	Mean (SD)	Range
Viewing personal health record	10.8 (9.8)	1-43
Viewing personal care plan	3.2 (3.2)	0-16
Messages to the care team	1.5 (2.0)	0-9
Viewing vaccination record	1.3 (1.5)	0-7
Prescription renewal	0.3 (0.6)	0-3

### Patient Portal Effect on Patient Activation

In analysis of variance, no significant effect of access to patient portal on patient activation was detected (*F*_1,133_=1.87, *P*=.17, η^2^=0.01). The mean activation score increased by 1.05 (SD 12.61), from 63.74 (SD 15.37) to 64.79 (SD 15.20), in the intervention group and decreased by 1.58 (SD 13.71), from 63.39 (SD 14.51) to 61.80 (SD 14.17), in the control group. The group difference at follow-up adjusted for baseline activation score was 2.77 (95% CI -1.24 to 6.79). As the difference of 4 to 5 points in patient activation is considered meaningful in terms of patients’ health behavior [26,27], the adjusted difference is minor.

### Main Effect of Baseline PAM Level on Change in PAM Score

**Figure 3:**
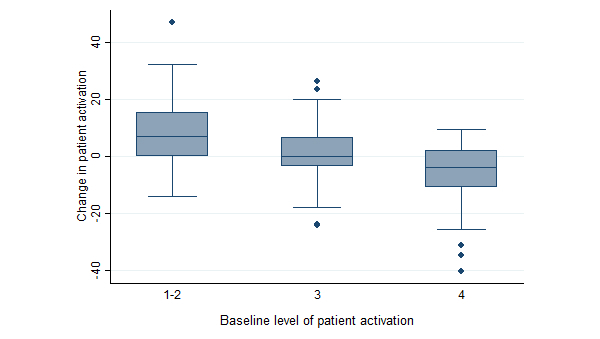
Changes in patient activation scores within groups starting at different levels of patient activation (n=137).

The 1-way analysis of variance showed a significant difference in mean change in patient activation score across the 3 groups starting from different levels of patient activation (*F*_2,137_ =17.90, *P*<.001, η^2^=0.21). Patients starting at low levels of patient activation (1-2) demonstrated greater positive change (mean change 8.5, SD 12.3) in activation score than patients starting at level 3 (mean change 0.7, SD 11.7) and 4 (mean change –6.1, SD 11.3; Figure 3). Pairwise post hoc comparisons using the Tukey HSD test further supported the statistical significance of the differences. Patients starting at the combined level 1-2 had significantly greater mean change scores than patients starting at level 3 (mean difference in change 7.8, *P*=.01) and level 4 (mean difference in change 14.6, *P*<.001). Furthermore, patients starting at level 3 showed a significantly greater mean change in patient activation than patients starting at level 4 (mean difference in change 6.8, *P*=.01). The difference of 4 to 5 points in patient activation is considered meaningful in terms of patients’ health behavior [26,27] and, thus, the differences between groups are considerable.

### Interaction Effect of Portal Access and Baseline PAM Level on Change in PAM Score

**Figure 4:**
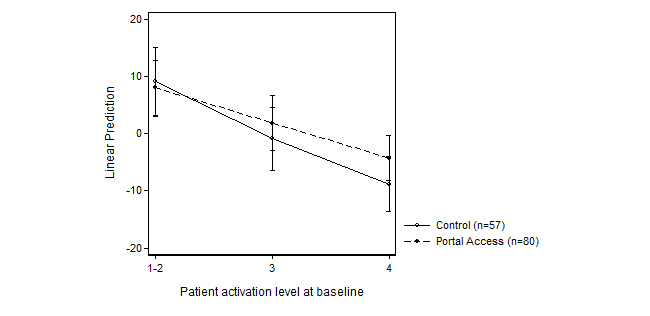
Intervention and control group estimates for mean change in patient activation (0-100 points) at different baseline levels of patient activation.

No statistically significant interaction effect on change in patient activation was detected between portal access and baseline PAM level (*F*_2,137_=0.62, *P*=.54, η^2^=0.009). Figure 4 presents the linear regression estimates (linear predictions) for change in patient activation by patient activation level at baseline. The most notable difference between the intervention and control groups was among patients starting from the highest level of patient activation (marginal effect=4.82, *P*=.13; Figure 4).

### Main Effect of Time Since Last Diagnosis on Change in PAM Score

**Figure 5:**
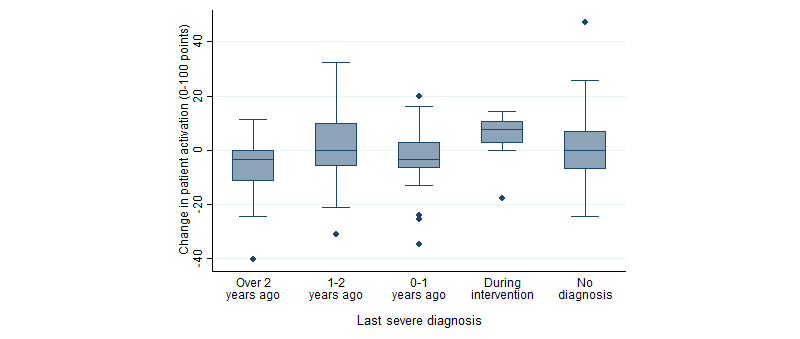
Changes in patient activation scores by time since last severe diagnosis (n=137).

The 1-way ANOVA revealed a significant difference in mean change in patient activation scores across the 5 groups with different temporal proximity of a severe diagnosis (*F*_2,137_=17.90, *P*<.001, η^2^=0.21). Patients diagnosed with a severe diagnosis during the intervention showed greatest positive change in patient activation (mean change 5.4, SD 8.4). In addition, patients diagnosed 1-2 years ago (mean change 2.3, SD 15.7) and patients with no severe diagnoses (mean change 1.6, SD 13.1) showed a positive change in patient activation. The greatest decrease in patient activation change was observed for patients with a severe diagnosis made more than 2 years before the intervention (mean change –7.1, SD 12.3), and the change was also negative for patients diagnosed less than 1 year before the intervention (mean change –3.0, SD 11.5), as shown in Figure 5. Pairwise post hoc comparisons using the Tukey HSD test showed a significant difference between patients diagnosed with a severe condition more than 2 years before the intervention and patients diagnosed during the intervention (mean difference in change 12.4, *P*=.02). The differences between the other groups were not statistically significant.

### Interaction Effect of Portal Access and Time Since Last Diagnosis on Change in PAM Score

**Figure 6:**
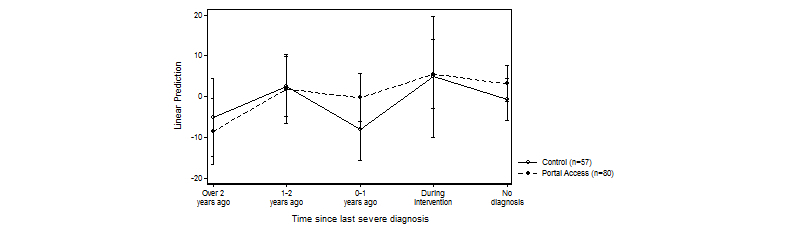
Group estimates for mean change in patient activation (0-100 points) in groups with different time since last severe diagnosis.

No statistically significant interaction effect on change in patient activation was detected between portal access and baseline activation level (*F*_2,137_=0.62, *P*=.64, η^2^=0.02). Figure 6 presents the linear regression estimates (linear predictions) for change in patient activation by time since diagnosis. The most notable difference between the intervention and control groups was observed among patients diagnosed within a year of the intervention (marginal effect=7.65, *P*=.12; Figure 6).

## Discussion

### Principal Findings

The psychometric assessment of the translated Finnish PAM13 instrument supported the reliability of the measure, and replicated to a great extent the findings from the previous Danish [24] and Dutch [25] versions. However, some rephrasing of question 12 may be required because more than 10% of participants scored the item as “not applicable” at follow-up.

No significant effect of access to patient portal on patient activation was detected in this study unlike previous research [9,10]. The heterogeneous results may be because of the different sets of functionalities provided through the portals studied. Nagykaldi and colleagues’ cluster randomized controlled trial [9] and Solomon and colleagues’ randomized controlled trial [10] both found a significant positive effect of an electronic patient portal on patient activation. Unlike the patient portal studied here, the interventions included features for patient-produced information of their health management [9] and interactive condition-specific health education [10]. The patient portal in the present study was relatively simple, so the addition of a greater number of activating functionalities might fortify its effect.

As has been observed in previous studies, the change in patient activation was greater among patients starting at a lower level of activation [10]. This pattern was similar in the intervention and control groups; thus, the effect of access to the patient portal was not greater for less-activated patients entering the intervention. In fact, the comparison of activation change between the intervention and control groups revealed that the positive effect of the intervention was greater among the patients starting at a higher level of patient activation. In the present study, the regression toward the highest score in the entire sample might be because of the additional intervention delivered to both the intervention and control groups, namely the drafting of the care plan. Another explanation could be the patient activation survey itself, in that it might encourage patients to rethink their role in the management of their condition. The latter alternative would call for the use of control groups to distinguish between the survey instrument effect and the actual intervention effect on changes in patient activation.

To our knowledge, this is the first study to examine the effect of time since diagnosis on patient activation. In both the intervention and control groups, a greater positive change in patient activation was identified among patients diagnosed with a severe condition during the intervention than among patients whose last severe diagnosis was made more than 2 years ago. This suggests that a severe diagnosis may have an independent immediate effect on patient activation. The intervention, in turn, appears to have made the greatest impact on the group diagnosed with a severe condition up to 1 year before the intervention. Among the few studies addressing the effect of time since diagnosis on care outcomes other than patient activation are those by Karter and colleagues [28], Blaum and colleagues [29], and van den Arend and colleagues [30], which were all conducted among diabetic patients. Karter and colleagues [28] found a connection between time since diagnosis and adherence to self-monitoring of blood glucose levels among diabetic patients by comparing patients diagnosed less than and more than 10 years ago, although the connections were inconsistent by diabetes type and severity of disease. Blaum and colleagues [29] discovered that time since type 2 diabetes diagnosis was longer for a group of patients with poor glycemic control compared with a group of patients with good control suggesting that the care outcomes may deteriorate as time since diagnosis increases. Van den Arend and colleagues [30] compared 4 different primary care programs for structured care of diabetes, and found that the longer patients had the diagnosis, the less their disease knowledge increased as a result of the programs. The somewhat incoherent results of the associations between time since diagnosis and different care outcomes suggest that the association may be dependent on the type of diagnosis, the outcomes measured, and the type of the performed self-management intervention. Understanding the effect of temporal proximity of a diagnosis may aid in identifying the sensitive periods in chronic care when “an exposure [to a specific chronic care intervention] has a stronger effect on development and hence disease risk than it would at other times” [31].

### Strengths and Limitations

The main strengths of this study are the experimental setting with longitudinal design and the use of scientifically validated measures for assessment of patient activation (PAM13) as well as in defining time since diagnosis (CCI).

As in any study, there are also several limitations. Three main limitations are related to the natural experimental setting. First, because the recruitment of the patients was conducted by clinical professionals and the time period for recruitment was limited, the sample size remained modest, reducing the statistical significance of the effects. The second limitation concerns the allocation of the patients in the intervention and the control groups. Although birth date itself is not expected to affect the outcomes of the intervention, the allocation method is deterministic in the sense that the assigned intervention could be predicted before the allocation [32]. This may have influenced the recruitment of the patients and may have contributed to the attrition imbalance between the intervention and control groups. Another likely reason for the attrition imbalance that could not have been avoided by randomization was the inability to blind the patients from receiving or not receiving access to the portal. An informed consent from the patients to participate was required for the ethical approval of the study. Therefore, the patients were aware of their assignment, which may have induced the greater dropout rate in the control group at the allocation phase (Figure 1). The third limitation of the study was the duration of the intervention. The intervention period of 6 months might have been too short to capture the full benefits of the portal. According to the professionals working in the study organization, both professionals and patients spent part of the intervention time learning how to effectively use the portal, despite the fact that a small scale pilot study with a restricted group of patients had been organized to test the portal before this investigation began. However, a longer intervention period would have been difficult to justify in a publicly funded health care organization, the central duty of which is to provide equal services to all its patients.

In this study, the participants formed a diagnostically heterogenic group. Because there may be differences in activation and its development in different diagnostic groups, further research is needed to assess the association of different diseases and patient activation. Furthermore, CCI, used in defining time since severe diagnosis, is restricted to a set of typical severe diagnoses; thus, some relevant diagnoses that might affect change in patient activation may possibly have been omitted. Broadening the set of diagnoses may further specify the relationship between time since diagnosis and patient activation.

### Conclusions

In this study, we created a Finnish translation of the validated PAM13 to evaluate the benefits of giving patients access to their medical records and secure messaging with health care professionals. Patient activation serves as “an intermediate outcome of care that is measurable and linked with improved [health] outcomes” [2].

No significant effect of a patient portal on patient activation was detected in this study. This result concerning a simple form of a patient portal differs from previous studies in which more interactive functionalities were included in the portal studied.

In addition to the functionalities offered through a patient portal, the activating effect of the portal is dependent on the characteristics of the patient who uses the portal. In this study, 2 patient-related factors, namely patient activation level at baseline and time since last severe diagnosis, were considered. Both variables were shown to be associated with changes in patient activation. Thus, it is suggested that these are considered in any evaluation of activating chronic care interventions. Further studies on the effect of time since diagnosis may identify sensitivity periods during which patients can benefit the most from specific chronic care self-management interventions. Findings on the factors affecting patient activation may aid in designing effective services for a heterogeneous group of patients with a distinct medical history and level of activation.

Patient portals are complex interventions in the way that their outcomes depend on multiple patient-related factors, such as recontacts with their health care provider during the intervention period, but also on the characteristics of the portal itself, such as the set of functionalities offered through the portal. We encourage further conceptual and empirical research on the mechanisms ignited by different patient portal functionalities and on the contextual factors that may moderate the effect of these mechanisms on patient outcomes.

## Multimedia Appendix 1

Patient Activation Measure.

## Multimedia Appendix 2

Charlson Comorbidity Index conditions.

## Multimedia Appendix 3

Data quality and item-rest correlations of the Finnish 13-item PAM (N = 137) at baseline and 6 months’ follow-up.

## Abbreviations

CCI: Charlson Comorbidity Index
HSD: honestly significant difference
PAM: Patient Activation Measure
