# Supplementary material for: The Effect of a Patient Portal With Electronic Messaging on Patient Activation Among Chronically Ill Patients: Controlled Before-and-After Study
Source: J Med Internet Res. 2014 Nov 19;16(11):e257. doi: 10.2196/jmir.3462 (PMC4260064; doi:10.2196/jmir.3462)
Supplement: Supplementary file 1 [file jmir_v16i11e257_app1.pdf]

Appendix 1. Patient Activation Measure.

Below are some statements that people sometimes make when they talk about their health. Please indicate how much you agree or disagree with each statement as it applies to you personally. Your answers should be what is true for you and not just what you think others want you to say.

1. When all is said and done, I am the person who is responsible for taking care of my health
2. Taking an active role in my own health care is the most important thing that affects my health
3. I am confident I can help prevent or reduce problems associated with my health
4. I know what each of my prescribed medications do
5. I am confident that I can tell whether I need to go to the doctor or whether I can take care of a health problem myself
6. I am confident that I can tell a doctor concerns I have even when he or she does not ask
7. I am confident that I can follow through on medical treatments I may need to do at home
8. I understand my health problems and what causes them
9. I know what treatments are available for my health problems
10. I have been able to maintain (keep up with) lifestyle changes, like eating right or exercising
11. I know how to prevent problems with my health
12. I am confident I can figure out solutions when new problems arise with my health
13. I am confident that I can maintain lifestyle changes, like eating right and exercising, even during times of stress

(Alternatives: Disagree Strongly, Disagree, Agree, Agree Strongly, N/A)
