# Supplementary material for: The Effect of a Patient Portal With Electronic Messaging on Patient Activation Among Chronically Ill Patients: Controlled Before-and-After Study
Source: J Med Internet Res. 2014 Nov 19;16(11):e257. doi: 10.2196/jmir.3462 (PMC4260064; doi:10.2196/jmir.3462)
Supplement: Supplementary file 2 [file jmir_v16i11e257_app2.pdf]

Appendix 1. Charlson Comorbidity Index conditions.

Myocardial infarction

Congestive heart failure

Peripheral disease

Cerebrovascular disease

Dementia

Chronic pulmonary disease

Connective tissue disease

Peptic ulcer disease

Mild liver disease

Hemiplegia

Moderate or severe renal disease

Diabetes with end-organ damage

Tumor

Leukemia

Lymphoma

Moderate or severe liver disease

AIDS
