# Supplementary material for: The Effect of a Patient Portal With Electronic Messaging on Patient Activation Among Chronically Ill Patients: Controlled Before-and-After Study
Source: J Med Internet Res. 2014 Nov 19;16(11):e257. doi: 10.2196/jmir.3462 (PMC4260064; doi:10.2196/jmir.3462)
Supplement: Supplementary file 3 [file jmir_v16i11e257_app3.pdf]

Appendix 2. Data quality and item-rest correlations of the Finnish 13-item PAM (N = 137) at baseline and 6 months' follow-up.

Baseline

Item	N	Mean PAM	SD	Median	% missing values	% not applicable	Item-rest correlation
1	137	3.39	0.65	3	0.00	2.19	0.32
2	136	3.46	0.62	4	0.73	0.73	0.44
3	137	3.29	0.66	3	0.00	2.92	0.43
4	137	3.08	0.78	3	0.00	5.11	0.57
5	137	3.22	0.70	3	0.00	4.38	0.54
6	137	3.30	0.75	3	0.00	2.92	0.47
7	136	3.57	0.68	4	0.73	5.11	0.50
8	137	3.32	0.71	3	0.00	2.92	0.62
9	137	3.14	0.73	3	0.00	5.11	0.71
10	137	2.83	0.80	3	0.00	2.92	0.49
11	137	3.17	0.66	3	0.00	3.65	0.61
12	137	2.87	0.73	3	0.00	9.49	0.73
13	136	2.70	0.82	3	0.73	5.84	0.54

6 months' follow-up

Item	N	Mean PAM	SD	Median	% missing values	% not applicable	Item-rest correlation
1	137	3.40	0.59	3	0.00	0.73	0.47
2	137	3.51	0.58	4	0.00	0.73	0.46
3	137	3.22	0.67	3	0.00	2.19	0.48
4	136	3.13	0.80	3	0.73	5.84	0.48
5	136	3.31	0.70	3	0.73	3.65	0.41
6	137	3.42	0.72	4	0.00	3.65	0.33
7	137	3.48	0.71	4	0.00	3.65	0.44
8	137	3.30	0.64	3	0.00	1.46	0.68
9	136	3.02	0.71	3	0.73	2.92	0.52
10	137	2.76	0.81	3	0.00	1.46	0.59
11	136	3.16	0.67	3	0.73	2.19	0.62
12	137	2.89	0.75	3	0.00	12.41	0.70
13	135	2.70	0.81	3	1.46	8.03	0.59
